# Integrated analysis reveals FLI1 regulates the tumor immune microenvironment via its cell-type-specific expression and transcriptional regulation of distinct target genes of immune cells in breast cancer

**DOI:** 10.1186/s12864-024-10174-9

**Published:** 2024-03-06

**Authors:** Jianying Pei, Ying Peng, Kexin Ma, Chunyan Lan, Tingting Zhang, Yan Li, Xiaofang Chen, Huafang Gao

**Affiliations:** 1 National Research Institute for Family Planning, Beijing, 100081 China; 2 Chinese Academy of Medical Sciences & Peking Union Medical College, Beijing, 100730 China; 3 Institute of Clinical Medicine, Gansu Provincial Maternity and Child-care Hospital (Gansu Provincial Central Hospital), Lanzhou, 730000 China; 4 Department of General Surgery, Peking University Third Hospital, Beijing, 100191 China; 5 Medical College of Northwest Minzu University, Lanzhou, 730030 China; 6 School of Biological Science and Medical Engineering, Beihang University, Beijing, 100083 China

**Keywords:** Breast cancer, FLI1, scRNA-seq, Immune cells, Transcription factor, Cell communication

## Abstract

**Background:**

Immunotherapy is a practical therapeutic approach in breast cancer (BRCA), and the role of FLI1 in immune regulation has gradually been unveiled. However, the specific role of FLI1 in BRCA was conflicted; thus, additional convincing evidence is needed.

**Methods:**

We explored the upstream regulation of FLI1 expression via summary data-based Mendelian randomization (SMR) analysis and ncRNA network construction centering on FLI1 using BRCA genome-wide association study (GWAS) summary data with expression quantitative trait loci (eQTLs) and DNA methylation quantitative trait loci (mQTLs) from the blood and a series of in silico analyses, respectively. We illuminated the downstream function of FLI1 in immune regulation by integrating a series of analyses of single-cell RNA sequence data (scRNA-seq).

**Results:**

We verified a causal pathway from FLI1 methylation to FLI1 gene expression to BRCA onset and demonstrated that FLI1 was downregulated in BRCA. FLI1, a transcription factor, served as myeloid and T cells’ communication regulator by targeting immune-related ligands and receptor transcription in BRCA tissues. We constructed a ceRNA network centering on FLI1 that consisted of three LncRNAs (CKMT2-AS1, PSMA3-AS1, and DIO3OS) and a miRNA (hsa-miR-324-5p), and the expression of FLI1 was positively related to a series of immune-related markers, including immune cell infiltration, biomarkers of immune cells, and immune checkpoints.

**Conclusion:**

Low-methylation-induced or ncRNA-mediated downregulation of FLI1 is associated with poor prognosis, and FLI1 might regulate the tumor immune microenvironment via a cell-type-specific target genes manner in BRCA.

**Supplementary Information:**

The online version contains supplementary material available at 10.1186/s12864-024-10174-9.

## Background

As the most frequently diagnosed malignancy, breast cancer (BRCA) heavily threatens the wellness and health of women worldwide. As recently reported, BRCA alone accounts for 31% of the total diagnosed cancers in women, and BRCA is the second leading cause of cancer death in women [1]. Due to its high heterogeneity, BRCA harbors many molecular subtypes, leading to various treatment options for this disease [2, 3]. The prognosis of BRCA patients is primarily related to the molecular subtypes, and almost all patients who develop the metastatic disease succumb to it. Thus, identifying the molecular mechanism contributing to BRCA progression or prognosis prediction is urgently needed.

Friend leukemia virus integration 1 (FLI1) is a transcription factor containing an ETS DNA-binding domain [4, 5]. FLI1 undergoes translocation with the Ewing sarcoma gene, thus leading to a fusion gene driving Ewing sarcoma by either transcriptionally inducing or repressing specific target genes, such as the RAS antagonist Sprouty 1 [6], E2F [7], and NOTCH-activated p53 [8]. The role of FLI1 in cancer has been increasingly reported, and FLI1 is a predictor of poor prognosis in patients with BRCA and promotes the metastasis and cancer stem cell properties of BRCA cells [9]. Conversely, FLI1 was also reported to be associated with shorter survival, and FLI1 downregulation in BRCA might promote tumor progression [10]. Several circRNAs consisting of FLI1 exons were identified as aberrantly expressed and are closely correlated with malignant phenotypes in small cell lung cancer and BRCA [11, 12]. Hence, the specific role of FLI1 in BRCA is conflicted, and we need more convincing evidence based on high-through data.

Immunotherapy is proving to be a practical therapeutic approach for various cancers. Tumor infiltration lymphocytes (TILs) have recently been acknowledged as a predictor or sensor for immunotherapy response prediction and monitoring to some extent in many tumors [13, 14]. For example, as measured by T-cell and myeloid cell infiltration, they convey inferior outcomes to conventional therapy compared with high immune infiltration [15]. The role of FLI1 in immune regulation and its potential as an immune target have gradually been revealed; for instance, CXCL13, a chemokine for B cells and regulatory T cells, is upregulated by FLI1 deficiency in macrophages, potentially contributing to the development of tissue fibrosis, vasculopathy and immune activation in system sclerosis [16]; FLI1 haploinsufficiency increases the proportions of Th2- and Th17-like Tregs in bleomycin-induced profibrotic skin conditions [17]; FLI1 was further reported to be related to many immune cell types and immune system processes in BRCA [18]. Thus, we speculated that FLI1 might play a crucial role in BRCA, especially in the immune regulation of BRCA.

In this study, we investigated the characteristics of FLI1 and possible regulatory mechanisms in BRCA via a combination of Mendelian random analysis and a series of bioinformatics analyses integrating BRCA GWAS, eQTLs/mQTLs data, scRNA-seq, and bulk-RNA-seq data. Our results implied that low-methylation-induced or ncRNA-mediated downregulation of FLI1 was correlated with poor prognosis, and that FLI1 might regulate the tumor immune microenvironment, primarily by targeting immune cells interactions, including myeloid cells and T cells, via cell-type-specific target genes in BRCA.

## Results

### The putative causal relationship of FLI1 in BRCA by integrating GWAS and eQTL/mQTL data

**Fig. 1:**
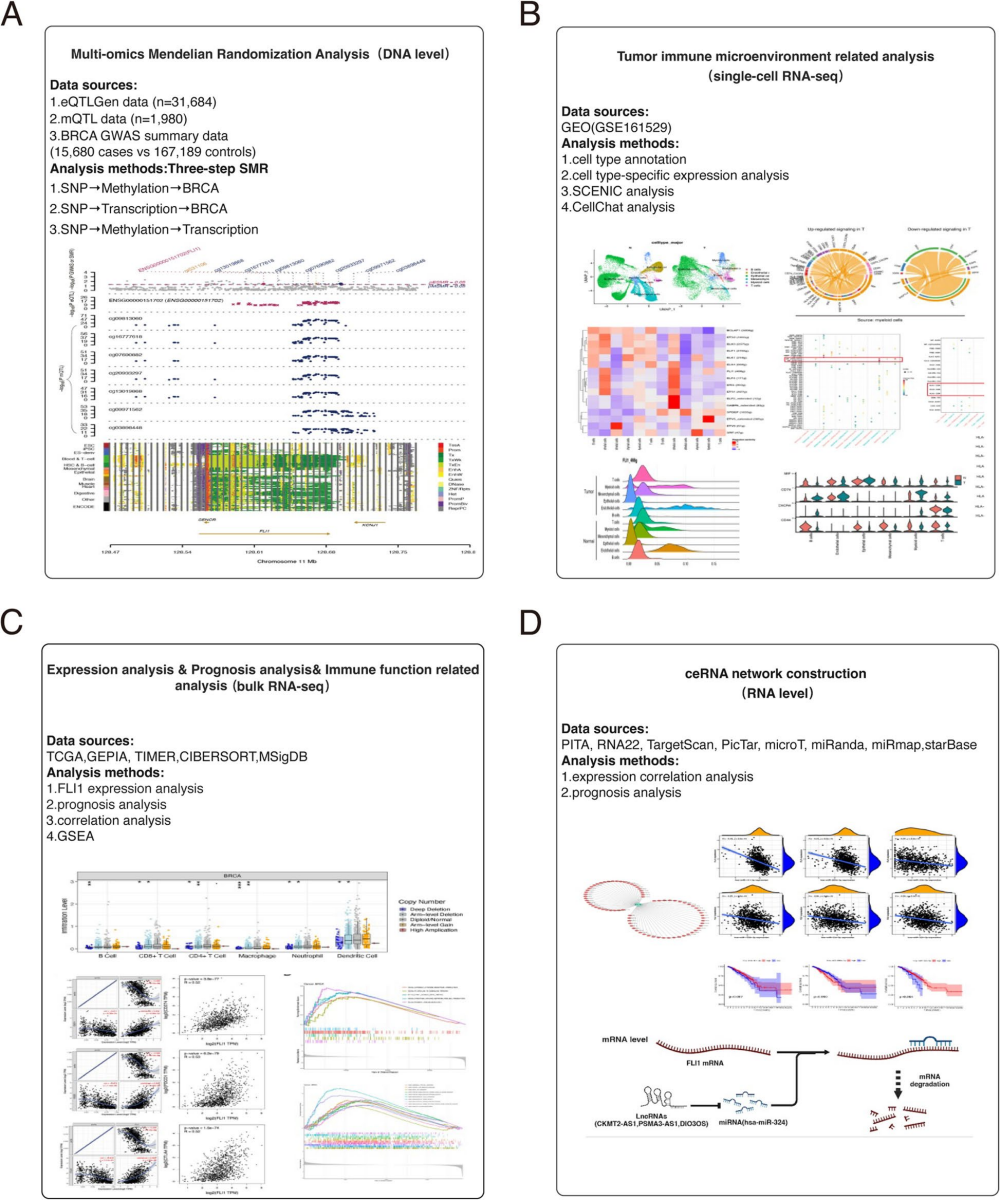
The workflow shows the strategy of this study

To explore the roles of FLI1 in tumorigenesis, we designed a series of analyses, as illustrated in Fig. 1. First, we explored the potential upstream epigenetic mechanism of FLI1 regulation in BRCA at the DNA level by using Mendelian randomization, which offers an alternative way to probe the issue of causality by using genetic variants [19] and combined with GWAS data and eQTL/mQTL data integrating gene expression and methylation omics data. Then, we applied scRNA-seq data of BRCA to investigate the role of FLI1 on the tumor microenvironment via integrating a series of single-cell analyses. Meanwhile, the bulk RNA-seq data of BRCA from the TCGA database was employed to unravel the correlation between FLI1 and immune-related markers, such as immune cell infiltration and immune genes. Besides the upstream regulation mechanism of FLI1 at the DNA level, we employed the bulk RNA-seq data and a series of ncRNA databases to construct a ceRNA network centered on FLI1 at the mRNA level. Finally, the expression of FLI1 in BRCA samples and its effect on the prognosis of BRCA patients were verified using TCGA data.

**Fig. 2:**
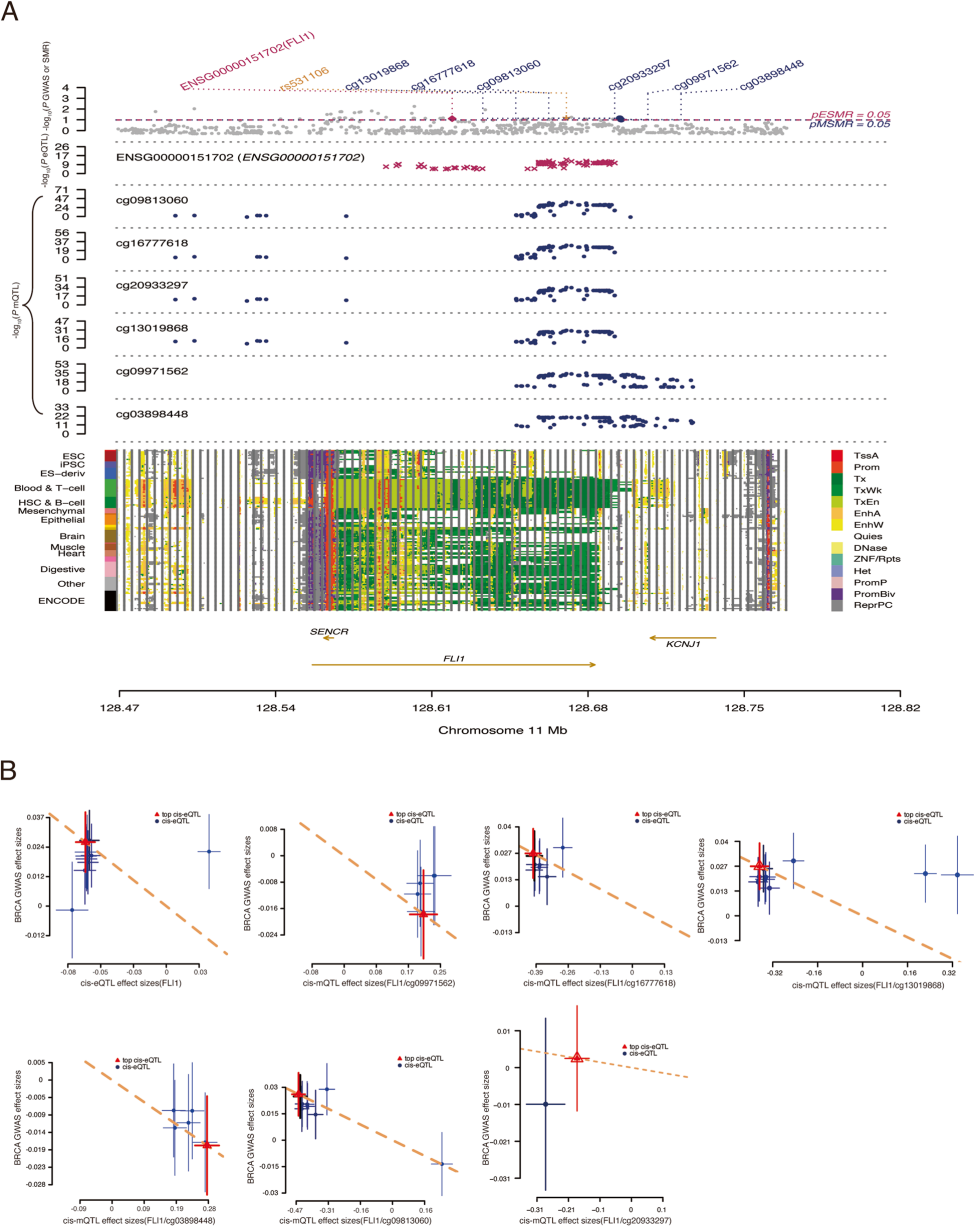
Three-step SMR analysis prioritized FLI1 and mechanisms in BRCA using blood tissue. **A** Locus zoom plots show the consistent genetic effects from the BRCA GWAS, cis-mQTLs, and cis-eQTLs near FLI1. The plot shows 14 chromatin state annotations (indicated by colors) of 127 samples from REMC for different primary cells and tissue types (rows). **B** SMR between FLI1 expression and BRCA GWAS (the first graph), SMR between FLI1 methylation (six methylation sites) and BRCA GWAS (the second to seventh graphs). REMC, Roadmap Epigenomics Mapping Consortium

To ascertain the causal relationship between FLI1 and BRCA, we utilized a three-step SMR analysis method, and FLI1-related cis-eQTLs and its cis-mQTLs were integrated with BRCA GWAS summary statistics. A total of 96 genetic proxies for the FLI1 gene (*P* < 5E-8) were obtained from eQTLGen (Supplementary Table 1), and we selected the top SNP, rs531106, for the first SMR analysis between FLI1 expression and the BRCA GWAS. The results demonstrated that FLI1 was indeed a BRCA-related gene (p_SMR < 0.05, p_HEIDI > 0.05, Supplementary Table 2). We then identified fourteen DNAm probes (within ± 1 Mb of the FLI1 gene) and corresponding SNPs by integrating mQTL summary statistics from a meta-analysis of two European cohorts, including the Brisbane Systems Genetics Study (BSGS, *n* = 614) and the Lothian Birth Cohorts (LBC, *n* = 1366). The second step SMR analysis results revealed that six DNA methylation probes were BRCA related (p_SMR < 0.05, p_HEIDI > 0.05, Fig. 2A & Supplementary Table 3). Finally, the last SMR step analysis revealed that all these six DNA methylation sites displayed a strong causal relation to FLI1 gene expression (p_SMR < 0.05, p_HEIDI > 0.05, Supplementary Table 4). Hence, our three-step SMR, determined by genetic variable instrument analysis, portraited an upstream mechanism from methylation to FLI1 expression further to BRCA. Specifically, the expression of FLI1 was negatively correlated to BRCA (Fig. 2B & Supplementary Fig. 1A), all six methylation sites of FLI1 were negatively correlated with BRCA, and all six methylation sites of FLI1 were positively correlated with FLI1 expression (Supplementary Table 4). We found that all six DNAm probes, including cg13019868, cg16777618, cg09813060, cg20933297, cg09971562, and cg03898448, were located in the same UTR region, 10 kb downstream of FLI1 (Supplementary Table 5 & Supplementary Fig. 1B). The methylation level of these sites had a positive effect on FLI1 expression (b_SMR > 0, Supplementary Table 4) and a negative effect on BRCA onset (b_SMR < 0, Supplementary Table 3); moreover, the expression of FLI1 was negatively associated with BRCA (b_SMR = -0.39, Supplementary Table 2).

### The cell-type-specific transcriptional regulatory role of FLI1 in BRCA

**Fig. 3:**
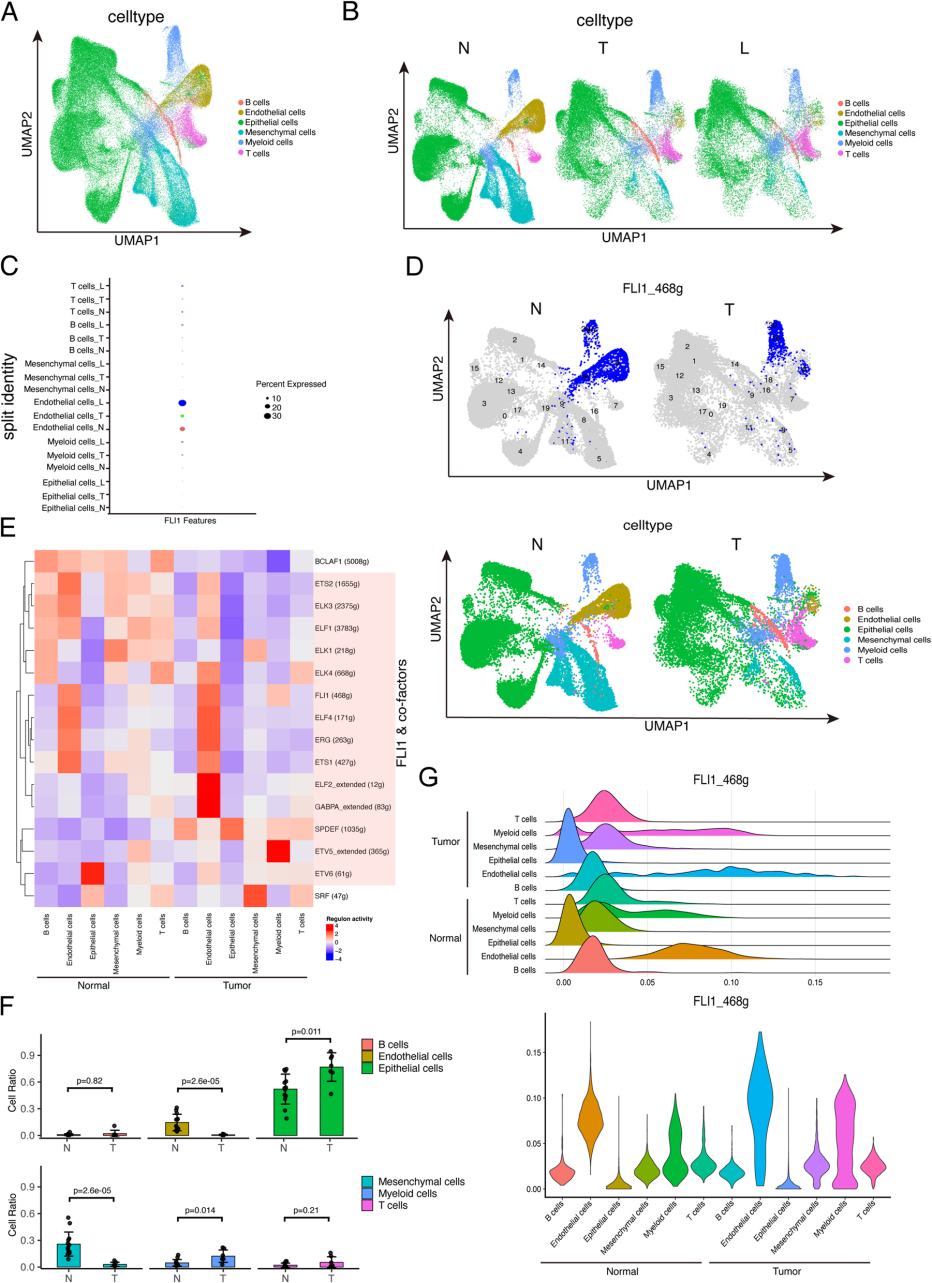
Single-cell transcription analysis and regulatory network centered on FLI1 in BRCA. **A** UMAP visualization of 99,532 cells analyzed by scRNA-seq and integrated across thirteen normal breast tissues, seven pairs of breast tumors, and corresponding lymph node samples, categorized into six major cell types. **B** UMAP visualization of all cells split by sample group. **C** The dot plot shows the expression of FLI1 in each cell type split by sample group. **D** UMAP visualization of the activity of FLI1 regulons in each cell type. **E** The heatmap shows the activities of the regulons of FLI1 and other cofactors in each cell type split by sample group. Colors from blue to red indicate low to high regulon activity (**F**). The ratio of each cell type in the normal and tumor groups. **G** Ridgeline plots and violin plots show the AUC values of the FLI1 regulatory network in each normal and tumor group cell type. N, normal. T, tumor. L, lymph node

To further analyze the detailed function of FLI1 in BRCA. We took part of the scRNA-seq data consisting of thirteen normal breast samples, seven pairs of breast tumor samples, and corresponding lymph node samples from a previous study (GSE161529) [20]. We downloaded the raw scRNA-seq data, processed the data as described in the Methods section, and ultimately acquired 99,532 cells, which were subjected to subsequent unsupervised graph-based clustering and visualized using uniform manifold approximation and projection (UMAPs). All 21 clusters were then manually annotated as six specific cell types using the expression of canonical lineage markers defined in previous literature (Fig. 3A & Supplementary Fig. 2A) [21, 22]. These cell populations consisted of endothelial cells (expressing PECAM1 and VWF), epithelial cells (expressing EPCAM, KRT18, and KRT8), B cells (expressing CD19, CD79A, and MS4A1), T cells (expressing CD2, CD3D, and CD3E), myeloid cells (expressing CD14 and CD68) and mesenchymal cells (expressing PDGFRB). All six cell types were dispersed in the normal, tumor, and tumor-involved axillary lymph node samples, but the specific ratio of each cell type differed among the groups. Among the normal samples, three major cell types were epithelial cells, endothelial cells, and mesenchymal cells, while among the tumor and tumor-involved axillary lymph node samples, the three major cell types were epithelial cells, myeloid cells and T cells (Fig. 3B).

We evaluated the expression of the FLI1 gene in a cell-type-specific manner and revealed that the expression of FLI1 varied among the different cell types (Fig. 3C). The FLI1 gene is strongly expressed in endothelial and immune cells, including myeloid cells and T cells, but is almost completely not expressed in epithelial cells and mesenchymal cells (Supplementary Fig. 2B). Additionally, we found an apparent trend that, regardless of cell type, the expression of FLI1 was largely augmented in tumor-involved axillary lymph node samples compared with normal breast tissues and BRCA tissues except mesenchymal cells (Supplementary Fig. 2C). As a typical transcription factor containing an ETS DNA-binding domain, FLI1 might regulate cell activity by targeting various downstream genes in a cell-type-specific manner. Hereafter, we utilized SECNIC, a computational method for cis-regulatory analysis and gene regulatory network reconstruction of transcription factors, using scRNA-seq data to determine whether FLI1 has distinctive downstream effects on different types of cells. The following analyses compared BRCA and normal tissues at the single-cell level. A total of 468 genes targeted by FLI1 were primarily activated in myeloid cells and endothelial cells in normal tissues but only in myeloid cells in tumor tissues (Fig. 3D). The regulons activity of FLI1 and most of its cofactors (ELF1, ELK1, ELK3, ELK4, ERF, ERG, ETS1, ETS2, ETV1, ETV2, ETV3, ETV4, FEV, ELF4, ETV3L, ETV7, SPDEF) indicated obvious enrichment in endothelial cells and immune cells (B cells, T cells, and myeloid cells) in normal tissues; however, those regulons activities of FLI1 and some cofactors (ELF1, ELK1, ELK3, ELK4, ETS2) was lost in tumor tissues (Fig. 3E & Supplementary Fig. 2D). The regulon activity of FLI1 was consistent with the cell-type expression pattern of FLI1, which exhibited high regulon activity in cells with high FLI1 expression (Supplementary Fig. 2E). In addition, we found that although the ratio of endothelial cells in tumor samples decreased compared with that in normal tissues, the regulon activity of FLI1 in endothelial cells in tumor samples was greater, and myeloid cells in tumors also displayed an apparent increase in the regulon activity of FLI1 (Fig. 3F, G). Accordingly, we speculated that myeloid cells is a targeted cell population via which FLI1 mainly exerted its regulatory effect on BRCA.

### The effect of FLI1 on cell–cell crosstalk by targeting immune-related ligands and receptors

**Fig. 4:**
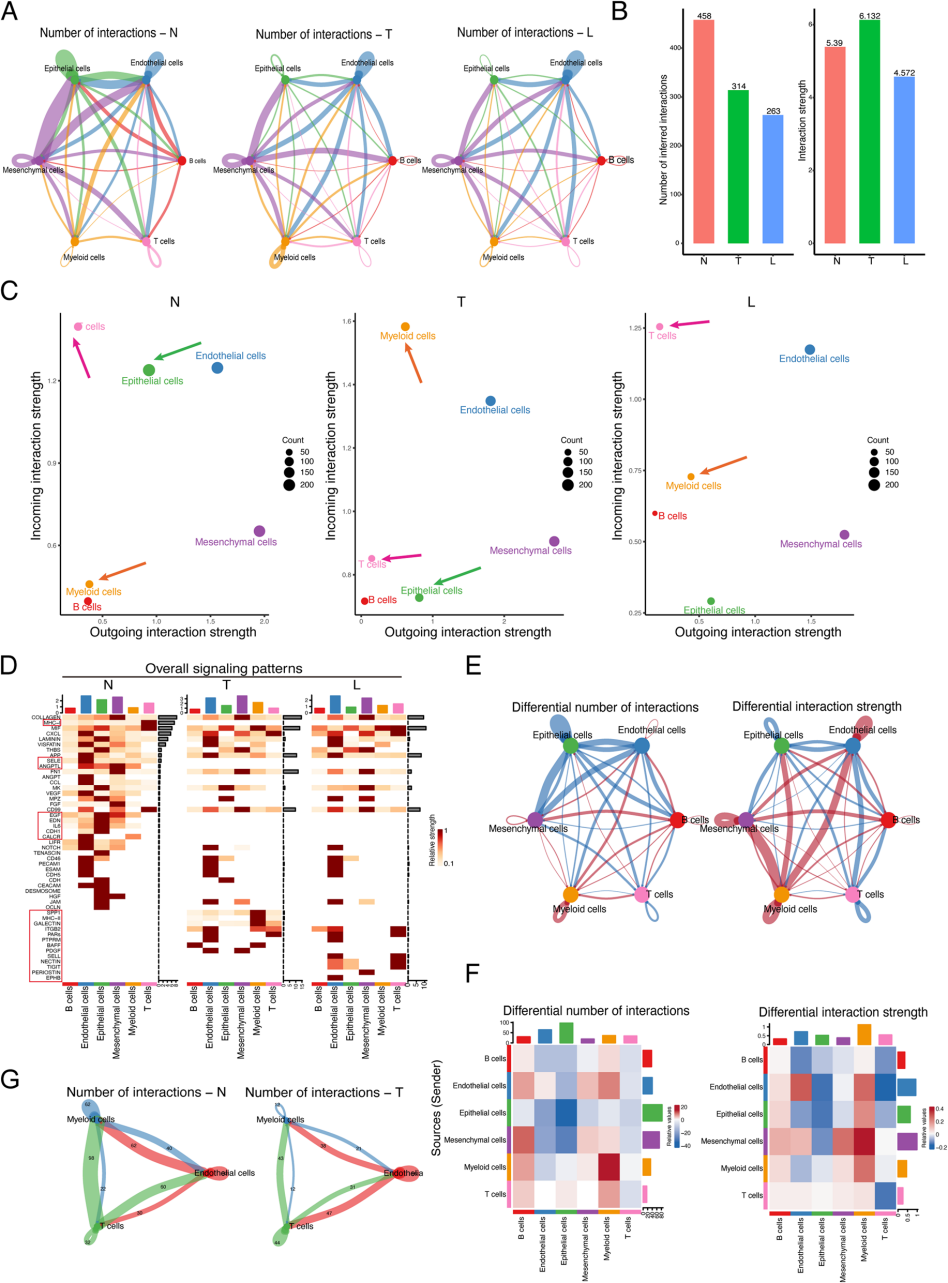
Global intercellular communication in BRCA. **A** Circle plots show the interaction numbers among all six cell types across different groups. **B** Bar plots show the total interaction number and interaction strength in different groups. **C** Scatter plots show the interaction strength of the incoming and outgoing of each cell type across different groups. **D** Heatmaps summarize the overall signal pathways of each cell group among all samples. **E** Differential number or strength of interactions in the tumor and normal groups. The red and blue lines indicate a signal increase and decrease in the tumor compared to the normal group. **F** Heatmaps show the differential number or strength of tumor interactions compared with the normal group. **G** The specific interaction numbers among myeloid cells, T cells, and endothelial cells in normal and tumor groups. N, normal. T, tumor. L, lymph node

**Fig. 5:**
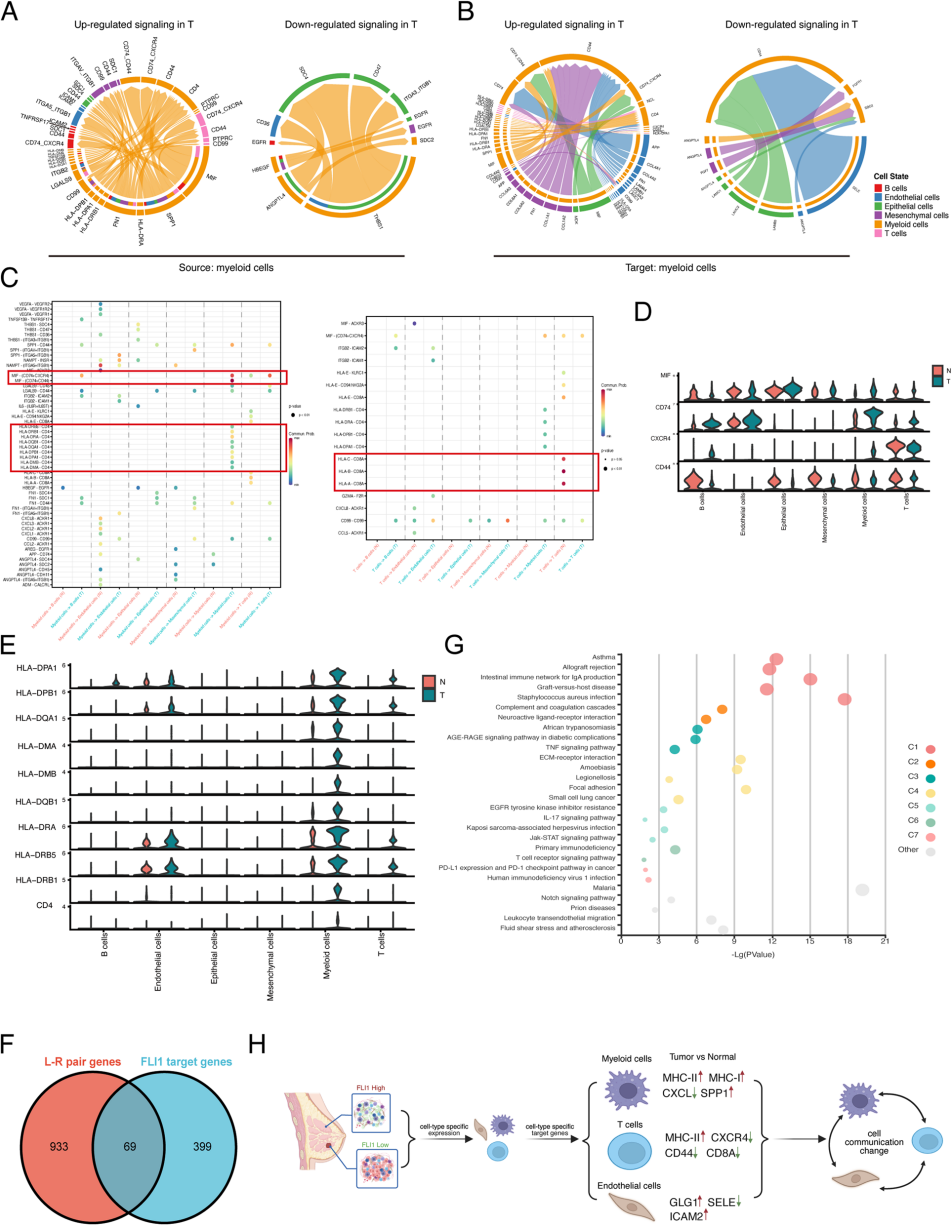
Crosstalk between myeloid cells or T cells and other cells in BRCA. **A**, **B** Circle plots show the up- or downregulated pathway of myeloid cells (**A**) or T cells (**B**) as source cells in communication with other cells in the tumor group compared with those in the normal group. **C** Bubble plots display the main signaling pathways mediating the cellular interaction from myeloid cells (left) or T cells (right) to other cells. **D**, **E** Violin plots show the detailed expression of the genes related to the MIF pathway (**D**) and the MHC II pathway (**E**) in each cell type in the different groups. **F** Venn plot of the intersection genes of FLI1 target genes and ligand-receptor genes in cellular communication. **G** Bubble plots presenting the KEGG analysis results of the intersection genes of FLI1 target genes and ligand-receptor genes. **H** A graph shows the potential mechanism by which FLI1 regulates immune cell interactions in BRCA

High heterogeneity in BRCA is always accompanied by sufficient intercell communication in the tumor microenvironment. Based on the above results that FLI1 exposed a high transcriptional regulation activity in myeloid cells, we further analyzed intercellular communication using CellChat, a tool that is able to quantitatively infer and analyze intercellular crosstalk networks from scRNA-seq data [23]. The results revealed that the number of global intercellular interactions decreased from normal samples to tumor samples and tumor-derived lymph node samples, while the interaction strength in tumor samples slightly increased (Fig. 4A, B). We compared the major sources and targets of intercellular communication signals in the different sample groups, and the results showed that the primary incoming signaling cell population changed from T cells in the normal group to myeloid cells in the tumor group; meanwhile, the position of epithelial cells in the global interaction network was greatly altered (Fig. 4C). We also discovered that in the tumor-derived lymph node samples, the signals of T cells restored the same position as those in the normal group. The myeloid cells, T cells, and epithelial cells displayed a relatively large degree of abnormal signal positions in the tumor compared to those in the normal group. The overall signaling pattern presented the major differential signal pathways, such as MHC-I, SELE, ANGPTL, IL6, CDH1, EGF, MHC-II, and SELL (Fig. 4D & Supplementary Fig. 3A, B). The main signaling pathways mediating intercellular communication in the normal and tumor groups are displayed in Supplementary Fig. 3C, and the specific signaling pathways of T cells and myeloid cells are shown in Supplementary Fig. 3D. A differentiation comparison between the normal and tumor groups revealed that crosstalks from myeloid cells to other cells increased regardless of the number or strength of interactions. In contrast, crosstalks from T cells to other cells decreased (Fig. 4E, F). The intercellular interactions of three major FLI1 target cell populations, myeloid cells, T cells, and endothelial cells, decreased (Fig. 4G). As a communication signal source or target cell population, myeloid cells exhibited upregulated signaling of MIF, SPP1, FN1, and HLA pathways in tumors and downregulated signaling of THBS1 and ANGPTL4 pathways in tumors (Fig. 5A, B). The changed signaling pathways of T cells as the communication signal source or target cell population were CD99, SELE, and HLA pathways in the tumor group compared with the normal group (Supplementary Fig. 4A, B). In tumors, the global cell communication activities of myeloid cells and T cells were manifestly upregulated and downregulated, respectively. The communication probability analysis of each cell type disclosed dysregulated ligand-receptor pairs of each cell type in the tumor tissues compared with those in the normal group. The most dysregulated ligand-receptor pair of myeloid cells was CD74-CD44/CXCR4, which might mediate stronger contact within myeloid cells, such as dendritic cells or macrophages. However, in T cells, loss of contact within T cells was observed, and most interactions were mediated by HLA-CD8 (Fig. 5C). Other dysregulated ligand-receptor pairs of endothelial, epithelial, or B cells also differed (Supplementary Fig. 4C). From these cell-type-specific effects of FLI1 on cellular function, as a typical transcription factor, FLI1 likely has special target genes in different types of cells. Several examples of genes belonging to the above-dysregulated cell communication pathways were identified as cell-type-specific target genes of FLI1. For instance, the expression of MIF, CD74, and CXCR4 were predominantly upregulated in myeloid cells, but CD44 was predominantly downregulated in myeloid cells (Fig. 5D); the expression of MHC-II molecules, including HLA-DRA, HLA-DPA1, HLA-DPB1, HLA-DQA1, HLA-DMA, HLA-DMB, HLA-DQB1, HLA-DRB5 and HLA-DRB were all activated mainly in myeloid cells of the tumor group (Fig. 5E); CXCL was most downregulated in cells except T cells (Supplementary Fig. 4D); CD8A was most downregulated in T cells (Supplementary Fig. 4E). We intersected the 468 target genes of FLI1 and 1002 ligand-receptor pairs genes and found 69 FLI1-target genes that were ligand-receptor genes (Fig. 5F & Supplementary Table 6). KEGG and GO analyses of these 69 genes revealed their roles in immune-related functions, for example, the IL-17 signaling pathway, primary immunodeficiency, the T cell receptor signaling pathway, PD-L1 expression, and the PD-1 checkpoint pathway in cancer, MHC-II protein complex binding, MHC-II receptor activity, and cytokine activity (Fig. 5G & Supplementary Fig. 4F). The potential mechanism of the effect of FLI1 on cell–cell crosstalk by targeting immune-related ligands and receptors is shown in Fig. 5H. The FLI1 is mainly expressed in endothelial cells, T cells, and myeloid cells in BRCA tissues and corresponding normal tissues. We deduced its cell-type specific expression manner might lead to a lower expression level in tumor tissues rather than normal tissues, which resulted from a dramatic decrease in the ratio of endothelial cells in tumor tissues compared with adjacent normal tissues. Moreover, FLI1, as a typical transcription factor, harbors different target genes in different cell types. Although FLI1 might be downregulated in tumor mass, it is exerted as an immune regulator by targeting immune cells’ receptors or ligands expression in tumor mass, further affecting the communication of cells infiltrated in the tumor. For instance, FLI1 in myeloid cells activated MHC-II molecules’ expression, which mainly improved the tumor antigen presentation, but in the T cells, FLI1 inhibited CD8A expressions, which might harm T cell activation. Collectively, we proposed that FLI1 could act as an immune regulator via its cell-type-specific expression and target genes; as for the specific roles of each cell type, we thought further research was needed.

### The relationship between FLI1 and immune-related functional markers in BRCA

**Fig. 6:**
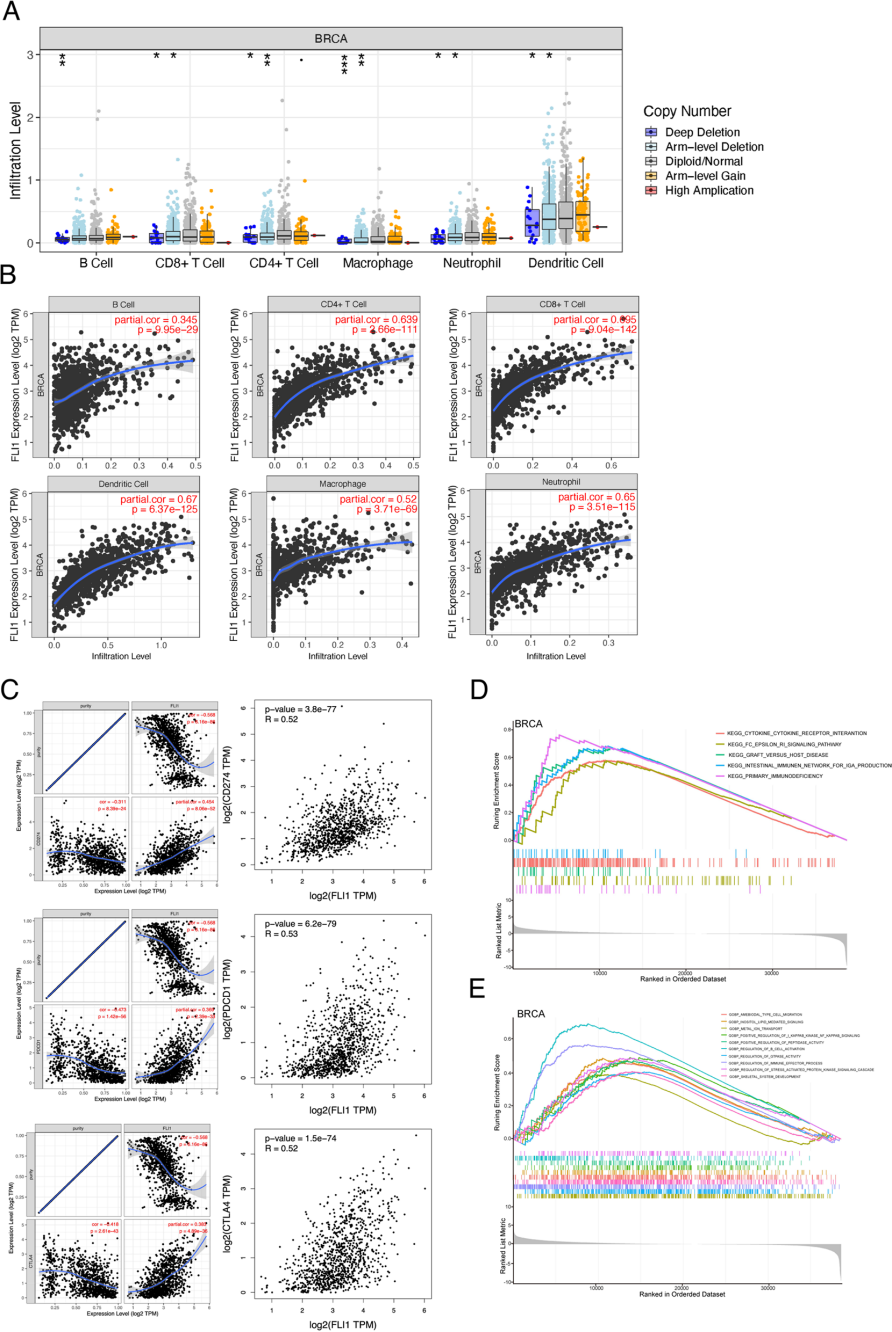
Correlation of FLI1 expression with immune-related markers in BRCA. **A** The infiltration levels of various immune cells with different copy numbers of FLI1 in BRCA. **B** The correlation of FLI1 expression level with B cell, CD4 + T cell, CD8 + T cell, dendritic cell, macrophage, or neutrophil infiltration levels in BRCA. **C** Spearman's correlation of FLI1 expression with PD-L1, PD1, and CTLA-4 expression in BRCA adjusted by purity using TIMER (left) and validated using the GEPIA database (right). **D**, **E** GSEA pathways (**G**) and GO biological process (**E**) enrichment analyses were performed using the single-gene method for FLI1in BRCA, and the top ten items were displayed

FLI1 was reported to participate in immune dysregulation in various diseases, including scleroderma and the tumor immune microenvironment in BRCA. Our study analyzed the relationship between the infiltration levels of various immune cells and the different copy numbers of FLI1 in BRCA. As shown in Fig. 6A, we found a strong correlation between deep deletion copy number or arm-level deletion copy number of FLI1 and the infiltration of almost all kinds of immune cells, namely B cells, CD8^+^ cells, CD4 + cells, macrophages, neutrophils, and dendritic cells. Moreover, we examined the association between the infiltration of immune cells and the expression of FLI1. The consequences stated that FLI1 expression was significantly positively associated with the infiltration levels of all the analyzed immune cells, including B cells, CD8^+^ cells, CD4 + cells, macrophages, neutrophils, and dendritic cells in BRCA, and most of these associations had relatively high correlation coefficients, which is greater than 0.5 (Fig. 6B). In addition, we analyzed the correlation of FLI1 expression with immune cell biomarkers using the GEPIA database. As shown in Supplementary Table 7, the expression of FLI1 was almost positively correlated with biomarkers of all types of immune cells. Some of them presented a relatively high correlation coefficient (> 0.5), such as biomarkers of B cells (CD19, CD79A), CD8^+^ T cells (CD8A, CD8B), CD4^+^ T cells (CD4), M2 macrophages (CD163, MS4A4A), Neutrophils (CCR7) and dendritic cells (HLA-DBP1, HLA-DQB1, HLA-DRA, HLA-DPA1, CD1C, NRP1, and ITGAX). We subsequently assessed the relationship between FLI1 and three typical immune checkpoint proteins, including PD-1, PD-L1, and CTLA-4. Considering the tumor purity of TGCA samples, we also performed the purity correction before correlation analysis, and the results revealed that FLI1 was also positively correlated with these three checkpoint molecules in BRCA in both the TGCA and TIMER databases (Fig. 6C). We further performed a gene set enrichment analysis of FLI1 expression in BRCA, and the results displayed that several immune-related pathways, such as cytokine receptor interaction, the intestinal immune network for IgA production, and primary immunodeficiency, were activated in patients with high FLI1 expression (Fig. 6D). Biological process analysis also found the activation of the NF-kappa signaling process, the regulation of B cell activation, and the immune effector process (Fig. 6E). Hence, FLI1 was hypothesized to be an indispensable key regulator of immune-related biological processes in BRCA.

### Prediction and construction of the upstream ncRNA network of FLI1

**Fig. 7:**
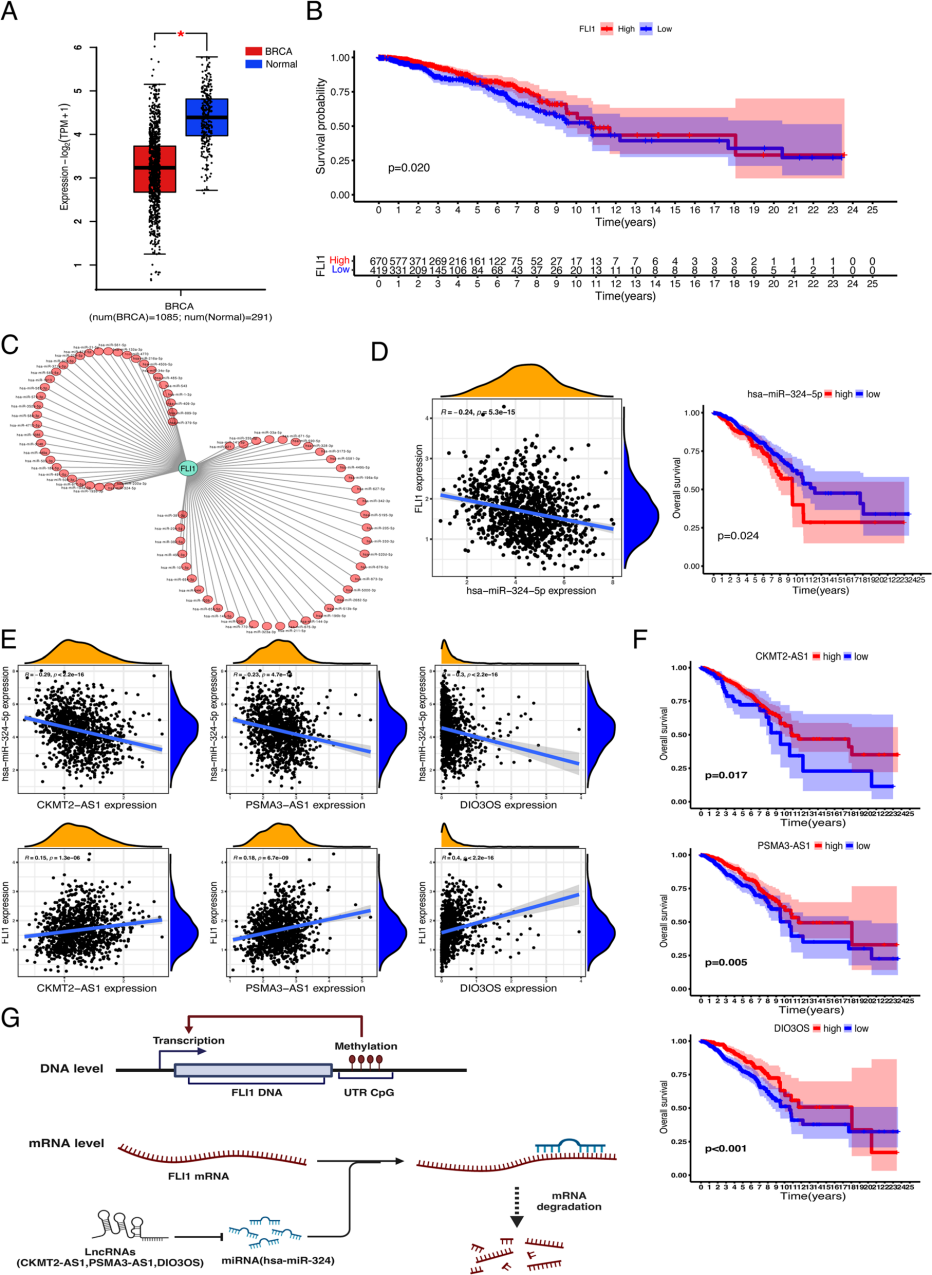
Establishment of the ceRNA network of FLI1 in BRCA. **A** FLI1 expression in BRCA tissues compared with corresponding TCGA and GTEx normal tissues. **B** Correlations between patients’ survival probability and FLI1 expression levels in BRCA. **C** The visible network of predicted miRNAs and FLI1 in BRCA constructed using Cytoscape. **D** Negative correlations between hsa-miR-324-5p and FLI1 and the prognostic value of hsa-miR-324-5p in BRCA. **E** Negative correlations between hsa-miR-324-5p and the predicted LncRNAs expression (upper panel), the positive correlations between FLI1 and predicted LncRNAs expression (lower panel) (**F**) The prognostic value of the candidate LncRNAs in BRCA. **G** The ceRNA network model of FLI1 in BRCA. *p value < 0.05; **p value < 0.01; ***p value < 0.001

Concerning the regulatory role of FLI1 in immune cell activity in the BRCA microenvironment, we found that FLI1 was significantly downregulated in BRCA tissues analyzed using bulk RNA-seq data derived from tumor tissues containing various cells (Fig. 7A). Meanwhile, we detected the relationship between the expression levels of FLI1 and patients’ prognosis and found that patients with higher expression of FLI1 in BRCA displayed better overall survival probability (Fig. 7B). These results indicated that FLI1 might be a protective factor in BRCA. Given that diverse upstream pathways might regulate FLI1, besides the methylation mechanism, we also focused on the potential regulatory network at the transcriptional level of FLI1. It has been broadly acknowledged that ncRNAs, as products of gene expression, regulate gene expression by affecting mRNA stability through direct or indirect binding. First, we predicted the upstream miRNAs that could bind to FLI1 mRNA using several target gene prediction databases. As shown in Fig. 7C, we constructed a network between FLI1 and its 103 potential upstream miRNAs (Supplementary Table 8). We found six miRNAs, including hsa-miR-141-3p, hsa-miR-200a-3p, hsa-miR-33b-5p, hsa-miR-193b-3p, hsa-miR-33a-5p and hsa-miR-324-5p, which were significantly negatively correlated with FLI1 in BRCA (Supplementary Fig. 5A, Supplementary Table 9). We further detected the expression of the six miRNAs in BRCA, and only hsa-miR-193b-3p presented non-differential expression between BRCA and normal tissues; the rest of the miRNAs exhibited significantly higher expression in BRCA than in normal tissues (Supplementary Fig. 5B). The prognostic values of these candidates reflected that only hsa-miR-324-5p was significantly correlated with prognosis prediction. A higher expression of hsa-miR-324-5p prophesied a poorer prognosis in BRCA patients, completely contrasting with the prognostic prediction ability of FLI1 (Fig. 7D & Supplementary Fig. 5C). Systematical combining the above results, we conjectured that hsa-miR-324-5p might be the most likely upstream miRNAs regulating the expression of FLI1 in BRCA.

In addition to miRNAs, LncRNAs are also crucial components of the competing endogenous RNA regulatory network, which can positively regulate targeted mRNA expression by competing for the same miRNAs with the targeted mRNAs. To establish the ceRNA regulatory network of FLI1, we primarily predicted the potential upstream LncRNAs of hsa-miR-324-5p using the starBase database. A total of 77 LncRNAs were identified, and expression correlation analysis between these candidate LncRNAs and hsa-miR-324-5p or FLI1 in BRCA were performed, respectively. Among all 77 LncRNAs candidates (Supplementary Table 10), we discovered three LncRNAs, CKMT2-AS1, PSMA3-AS1, and DIO3OS, which presented negative correlation and positive correlation with hsa-miR-324-5p expression and FLI1 expression, respectively (Fig. 7E, Supplementary Table 11). The expression levels of the three LncRNAs in BRCA were determined using GEPIA, and all of them were significantly downregulated compared with those in normal tissues, with the same expression trend observed for FLI1 (Supplementary Fig. 5D). Furthermore, all three LncRNAs could predict the prognosis of patients with BRCA (Fig. 7F). As shown in Fig. 7G, we proposed a potential upstream mechanism to regulate FLI1 transcription activity and FLI1 mRNA stability in BRCA. On the one hand, the methylation in the UTR CpG region of the FLI1 gene leads to its transcription activation. On the other hand, there is a ceRNA network centering on FLI1 mRNA stability, a miRNA termed has-miR-324 could bind to FLI1 mRNA to induce its degradation while three LncRNA, CKMT2-AS1, PSMA3-AS1 and DIO3OS, could compete with has-miR-324.

## Methods

### SMR data resources

The GWAS summary statistics of malignant neoplasms of the breast (controls excluding all cancers, ICD-O-3) were downloaded from the latest release R9 version database of the FinnGen research project (https://www.finngen.fi/en/access_results), which encompassed 15,680 cases and 167,189 controls [24]. Blood eQTL summary data of the FLI1 gene were SMR-formatted cis-eQTLs derived from the eQTLGen consortium (http://www.eqtlgen.org), which consists of 31,684 blood and PBMC samples from 37 individual cohorts [25]. Blood mQTLs summary statistics were obtained from a meta-analysis of two European cohorts, including the Brisbane Systems Genetics Study (BSGS, *n* = 614) and the Lothian Birth Cohorts (LBC, *n* = 1366) [26]. Our study focused only on cis-regulatory rather than trans-regulatory elements; thus, SNPs within ± 1 Mb of the FLI1 gene were selected for related analyses. All those mQTLs and eQTLs data were downloaded from the Yang Lab website (https://yanglab.westlake.edu.cn/software/smr/#mQTLsummarydata).

### SMR and HEIDI analysis

The SMR tool was utilized to examine the causal inference of FLI1 gene expression in BRCA occurrence. The 1000 Genomes European dataset was used as the reference for calculating linkage disequilibrium (LD). We conducted a three-step SMR analysis as follows: (1) blood FLI1 gene expression was exposure, and BRCA was the outcome, while the instrumental variable was the top SNP showing the strongest association with the FLI1’s expression among all 96 candidates SNPs (*P* < 5E − 8, Supplementary Table 1) [27]. (2) SNPs within ± 1 Mb of the FLI1 gene were selected as instruments, blood DNAm was exposure, and BRCA was the outcome. (3) SNPs within ± 1 Mb of the FLI1 gene were selected as instruments, blood DNAm was exposure and blood FLI1 gene expression was the outcome. In the SMR analysis, we utilized the default threshold of P eQTL = 5E − 8 to select the top associated cis-eQTL for the SMR analysis. We removed SNPs with allele frequency difference > 0.2 between any pairwise data sets, including the reference data 1000 Genomes European dataset), the eQTL summary data, the mQTL summary data, and the GWAS summary data. We also conducted the heterogeneity in dependent instruments (HEIDI) test to evaluate the existence of linkage. The final criteria to select positive signals were defined as follows: (1) SNPs showed significant genome-wide association in all eQTLs, mQTLs, and GWAS (*P* < 1E − 5). (2) SNPs passed all three-step SMR analyses (pSMR < 0.05). (3) SNPs presented heterogeneity in the HEIDI test of all three-step SMR analyses (pHEIDI > 0.05). Data curation and bioinformatical analysis were performed using R version 4.0.3 (https://www.rproject.org/), PLINK 1.9 (https://www.cog-genomics.org/plink/1.9/), and SMR (https://cnsgenomics.com/software/smr/).

### scRNA-Seq data collection and processing

The scRNA-Seq raw data used in this study were obtained from the Gene Expression Omnibus (GEO) database (www.ncbi.nlm.nih.gov/geo/, GSE161529). The sample information is presented in Supplementary Table 12, which includes thirteen normal breast samples, seven pairs of breast tumor samples, and corresponding lymph node samples composed of ER + tumor total cells and ER + tumor lymph node cells, respectively. The Seurat R package (version 4.2) was used to analyze scRNA-seq data, and doublets were identified using DoubletFinder (version 1.0.1) with default parameters [28, 29]. The following criteria were exploited for each sample: unique gene number > 500, unique molecular identifier (UMI) count > 1000, and mitochondrial gene percentage < 0.15. We merged the datasets and normalized the raw counts using the MergeSeurat and NormalizeData functions. High-variable genes were identified with the FindVariableFeatures function. We used the ScaleData function to scale and centralize datasets and regressed out UMI numbers, mitochondrial gene percentages, ribosomal gene percentages, and heat shock protein gene percentages. Subsequently, different ScRNA-seq datasets were integrated using the “integrated CCA” function in Seurat and dimensions of 1:30 to correct the batch effect.

### Dimensionality reduction and cell type identification

For dimensionality reduction, highly variable genes were used for principal component (PC) detection. Based on the expression table according to the UMI counts of each sample and percent of mitochondria rate, raw feature counts were log-normalized, scaled, and subjected to principal component analysis (PCA), which was performed based on the scaled data of the top 2000 highly variable genes. The top 10 PCs were used for UMAP construction, and unsupervised cell cluster results based on the top 10 PCs were obtained using a graph-based cluster method. Cells were subsequently clustered with a "resolution" set to 0.9, identifying 21 primary clusters. These clusters were then annotated according to the expression of lineage-specific genes in previous works. The marker genes using the FindAllMarkers function with the Wilcoxon rank sum test algorithm were calculated with the following criteria: (1) lnFC > 0.25; (2) *p*-value < 0.05; and (3) min.pct > 0.1. To identify specific cell types, clusters of the same cell type were selected for re-UMAP analysis, graph-based clustering, and marker analysis.

### Cell–cell communication and gene regulatory network analysis

Cellchat (version 1.1.0) was used to detect the inference and analysis of cell–cell communication with default parameters [23]. The versatile toolkit CellChat and a web-based Explorer (http://www.cellchat.org/) were used to build cell–cell communication atlases. To assess the regulatory strength of TFs, the SCENIC (version 0.9.5) workflow, a new computational method for the construction of regulatory networks and identification of different cell states from scRNA-seq data, was used using the 20 000 motif database for RcisTarget and GRNboost [30]. GENIE3 in SCENIC was employed to build gene regulatory networks using an expression matrix and select potential direct-binding targets (regulons) through DNA motif analysis. The activity of regulons in individual cells was scored by the AUCell function, and the regulon activity scores in each sample were scaled and calculated using the Limma R package (version 4.2) using the following criteria: adj. *P* value < 0.05 and |logFoldChange|> 0.25.

### GO and KEGG pathway analysis

The clusterProfiler package in R was selected to perform gene ontology (GO) functional annotation and Kyoto Encyclopedia of Genes and Genomes (KEGG) analyses to investigate and visualize the biological function of 69 FLI1 target genes. The Fisher exact test was used to identify the significant GO categories, and a false discovery rate (FDR) was used to correct the *p*-values. A *p*-value < 0.05 and an FDR < 0.05 were considered statistically significant.

### Bulk RNA-seq data collection and survival analysis

The transcriptome profiles (HTSeq-FPKM) of 1,222 samples, consisting of 1,109 breast tissues of patients with BRCA and 113 adjacent tissues, and corresponding clinical information were downloaded from the TCGA (https://genome-cancer.ucsc.edu/). Each sample's expression data or clinical information was combined into corresponding matrix files using Perl language. Ensemble IDs in the expression matrix profile were also converted into Gene Symbols with Perl language. ID numbers in the expression and clinical information profiles were matched, and samples whose ID numbers did not match were excluded from our study. Finally, we obtained 1,097 BRCA cases for subsequent analysis. Raw expression data were transformed with log2 and normalized for the following differential expression analysis of FLI1 using the limma package in R language [31]. *P* < 0.05 and FDR < 0.05 were considered statistically significant. GEPIA (http://gepia.cancer-pku.cn/) was used to verify the differential expression levels of FLI1, CKMT2-AS1, PSMA3-AS1, and DIO3OS in BRCA, which is an online web tool containing gene-expression profiles based on TCGA and The Genotype-Tissue Expression (GTEx) data [32]. *P* < 0.05 was considered statistically significant. The survival analysis of FLI1, candidates’ miRNAs, and LncRNAs in BRCA were all performed using TCGA data. We set the median expression values of FLI1 or candidates’ miRNAs and LncRNAs in all BRCA samples as the cut-off values. According to the cut-off values, the BRCA samples in TCGA were divided into the high-expression or the low-expression group, respectively. A log-rank test was performed to compare the differences in OS between high- or low-expression groups. The survival analysis between these two groups was conducted using the survival package in R software.

### Candidate miRNAs / LncRNAs selection and ceRNA network construction

Several target gene prediction databases, including PITA, RNA22, TargetScan, PicTar, microT, miRanda, and miRmap, were used to predict the upstream binding miRNAs of FLI1, and we chose the miRNAs that appeared in more than two databases’ prediction results as the candidate miRNAs of FLI1. The starBase database was exploited to predict the binding LncRNAs of has-miR-324-5p. The mutual expression correlations between FLI1 and miRNAs or LncRNAs were conducted using R language based on the expression data of 1097 BRCA samples in TCGA, and the ceRNA network was visualized using the Cytoscape software.

### Immune cell infiltration and checkpoint analysis

The immune cell infiltration for all BRCA tumor samples was downloaded from an online website named Tumor Immune Estimation Resources (TIMER) (https://cistrome.shinyapps.io/timer). Then, it was employed to complete the correlation analyses between FLI1 expression and the infiltrating levels of different subtypes of immune cells [33]. Specifically, the TPM data of RNA-seq was converted from FPKM data and used for estimating the abundance of different immune cell types by CIBERSORT (https://cibersort.stanford.edu/) [34]. We attained the immune cell types and their corresponding biomarkers gene lists from TIMER for the checkpoint analysis. Then, the expression values of each biomarker were extracted from the 1109 BRCA samples, and the correlations were analyzed. The limma, the cor. function, ggplot2, ggpubr, and ggExtra packages in R were utilized as needed in these analyses.

### Gene Set Enrichment Analysis (GSEA)

The 1097 BRCA samples of TCGA were divided into two groups according to the median expression value of FLI1, thus resulting in the high FLI1 group and low FLI1 group. GSEA was implemented to discover the gene sets enriched in the gene rank in the two groups to recognize the potential KEGG pathways and biological processes of FLI1 in BRCA. The annotated gene sets of h.all.v6.2.symbols.gmt in the Molecular Signatures Database (MSigDB) were selected in GSEA version 3.0. We executed 1,000 times of permutations. The collapse dataset to gene symbols was “False.” The permutation type was “phenotype.” GSEA was run, and the cut-off criteria were as follows: normalized enrichment scores (NES) > 1.0, false discovery rate (FDR) q > 0.25, and nominal *p* < 0.05. Then, we showed the top 10 KEGG pathways and biological processes.

## Discussion

Tumor-infiltrating lymphocytes (TILs), a population of immune cells infiltrating in tumor tissue, have been reported in various solid cancers, such as BRCA, colon, and lung cancer [35–37]. In recent decades, evidence of TILs as a prognostic biomarker, which came from clinic research containing thousands of BRCA patients, rapidly soared. For example, the triple-negative BRCA patients displayed a robust linear relationship between increased TILs and improved recurrence-free survival (RFS) consequences [38–40]. Hence, the definite correlation between TILs and antitumor therapy consequences triggered a series of studies focused on seeking novel immune targets that might enhance the TIL population by regulating immune-related processes in BRCA tissues and ultimately improve patients’ prognosis. With this goal, we designed this study. We focused on FLI1, which has been documented as a member of the ETS family with a crucial role in hematopoiesis and development pathways in both immune and non-immune cells [41, 42]. To comprehensively investigate the correlation between FLI1 and BRCA, we investigated the latent upstream regulatory mechanisms of FLI1 expression at both the DNA and RNA levels.

First, we clarify the causal relationship between FLI1 and BRCA using the currently available BRCA GWAS and FLI1 mQTL/eQTL data, which were analyzed via the Mendelian randomization method. The causal pathway linking CpG methylation of FLI1 to FLI1 mRNA expression and finally to BRCA susceptibility was identified. A three-step SMR analysis verified a negative correlation between the mRNA expression and the BRCA onset, a positive correlation between the methylation of FLI1 and its mRNA expression, and a negative correlation between the methylation of FLI1 and the BRCA onset. The traditional view is that a certain gene's high DNA methylation always leads to repressed transcription activity [43]. However, recent years’ work from various models indicated that hypermethylated promoters and enhancers could be permissive to the transcription of a certain gene rather than a dominant repressive mechanism [44]. In our study, we found higher methylation of FLI1 positively related to its expression, meanwhile negatively related to BRCA occurrence risk. Thus, we speculated that the methylation of FLI1 might be one of the mechanisms affecting BRCA via its regulation of FLI1 gene expression. Besides methylation at the DNA level, ncRNAs are another significant regulator of target gene expression by interacting with each other through the ceRNA network. We thus constructed a potential ceRNA network targeting FLI1 at the RNA level mainly based on expression correlation and prognosis correlation analyses. Furthermore, we elucidated the downstream regulatory mechanism of FLI1 in BRCA relying on bulk-RNA seq data and single-cell RNA-seq data. On the one hand, the FLI1 expression strongly correlated with immune-related markers, including immune cell infiltration, immune cell marker genes, and immunotherapy checkpoint genes. On the other hand, we found the cell-type-specific expression and function of FLI1 in BRCA based on single-cell analysis, especially showing cell-type-specific target genes and regulating intercellular communication in tumor microenvironment in BRCA. We verified the differential expression of FLI1 in BRCAs. We found FLI1 was downregulated in BRCA tissues compared with normal tissues, and BRCA patients with higher expression levels of FLI1 showed better overall survival probability. These results were also consistent with the previous study [18].

The functions of FLI1 in regulating immune cell development, activation, migration, and exhaustion have been gradually discovered. It can also affect the function of immune cells by regulating cytokines and chemokines [45]. Thus, we demonstrated a positive relationship between FLI1 expression and immune cell infiltration or immune cell biomarker expression in BRCA, which indicated that one of the reasons why patients with higher expression levels of FLI1 had a better prognosis might be the abundant activation of the immune process in the tumor environment. Additionally, there is a positive correlation between FLI1 and immune checkpoint proteins, including PD-L1, PD1, and CTLA-4 in BRCA. It was reported that the circulating immune cells, namely PBMC, manifested differential gene expression profiles between responders and non-responders of anti-PD-1 therapy in melanoma patients [46]. Given the dominant expression in immune cells of FLI1, we thought it is worthy of further study to investigate the relationship between FLI1 and anti-PD-1 therapy, especially the different target genes of FLI1 in different immune cell populations, which might mediate the FLI1’s function in immunotherapy efficacy. The potential of FLI1 as a therapeutic target in human autoimmune diseases such as systemic sclerosis and systemic lupus erythematosus has been proposed, and the loss of FLI1 in CD8^+^ T cells enhances immunity to tumors has also been reported [41, 45]. The relationship between FLI1 and immune infiltration profiles has also been analyzed in a previous study based on resources from available databases such as the TCGA [18].

To unveil the specific molecular mechanisms by which FLI1 regulates immune-related processes in BRCA. We utilized scRNA-seq data, which could provide more information than bulk-RNA sequencing, especially for highly heterogeneous cancers. Surprisingly, we found that FLI1 is expressed mainly in endothelial cells, moderately in immune cells, including myeloid cells, T cells, and B cells, but hardly in epithelial cells. Accordingly, immune function regulation by targeting immune cells infiltrating the tumor microenvironment might be a crucial link between FLI1 and BRCA. As a transcription factor, FLI1 could activate or inhibit the expression of a series of target genes by binding to their transcription regulation region. Subsequent analyses of SECNIC demonstrated that the transcriptional activity of FLI1 indeed varied in different cell types. Endothelial cells and myeloid cells in tumor tissues exhibit high FLI1 expression and high transcription activity of FLI1-targeted genes. We found evident activation of FLI1-targeted genes in myeloid cells of tumor samples compared with those of normal tissues.

Further analyses of cellular communication revealed that, in tumor samples, the communication between intratumor cells was different from that in normal tissues, and target genes of FLI1 showed a large intersection with ligand or receptor genes mediating cellular crosstalk. We discovered several cell-communication-related target genes of FLI1 in immune cells, including myeloid cells and T cells, that showed differential expression between tumor and normal tissues. For example, the obvious upregulation of MHC-II genes (HLA-DRA, HLA-DPA1, HLA-DPB1, HLA-DQA1, HLA-DMA, HLA-DMB, HLA-DQB1, HLA-DRB5 and HLA-DRB), CD74, and CXCR4 in myeloid cells of the tumor group was accompanied by a high probability of communication between cells in the population of intratumoural myeloid cells. MHC-II is constitutively expressed on a subset of cells termed professional antigen-presenting cells (APCs), including myeloid cells, which could further be classified into macrophages or dendritic cells. The enhanced expression of MHC-II is an important way for APCs to proliferate in response to pathogens [47]. CD74 participates mainly in antigen presentation as an MHC class II chaperone, and stromal CD74^+^ cell enrichment has been associated with favorable prognosis in patients with HCC [48]. The decreased expression of CD8A in T cells in the tumor group concurred with the decreased probability of communicating with B cells and T cells or with the cell population of intra-T cells. Collectively, we proposed that FLI1 functions as a BRCA protective factor likely via its transcriptional activation or inhibitory effect on a series of target genes in a cell-type-specific manner, especially ligand and receptor genes mediating intercellular communication in myeloid cells.

The ceRNA networks centered on FLI1 have rarely been reported, and most have been characterized in Ewing sarcoma. Several miRNAs, such as let-7g, miR-22, miR-30a-5p and miR-145, are closely related to EWS-FLI1 regulation [49, 50]. A ceRNA network targeting FLI1 has also reported in other types of cancers, including the miR-33b/FLI1 axis in hepatocellular carcinoma and the miR-145/FLI1 axis in colon cancer [51, 52]. There are no ceRNA network reports of FLI1 in BRCA. Our study combined various databases to navigate the potential upstream ncRNAs of FLI1 by combining the binding motif prediction and negative expression relationships. Consequently, we established a novel ceRNA network focused on FLI1 in BRCA based on the high-throughput sequencing data and the direct binding relationships between hsa-miR-324-5p and FLI1 or CKMT2-AS1, PSMA3-AS1, and DIO3OS need to be confirmed through other experiments in molecular biology.

This study still has certain limitations that need to be solved to decipher the explicit role of FLI1 in BRCA. First, in the SMR analysis, the methylation and expression data of FLI1 in the mQTL and eQTL were derived from blood samples rather than from breast tissues. Second, the ceRNA network of FLI1 was constructed using TCGA bulk-RNA sequencing data. However, we could not validate the competing mechanism using the subsequent scRNA-seq data owing to the technical defects. Third, we did not further categorize the immune cells into tumor or normal groups because the total cell number of immune cells did not support further classification or subsequent cellular communication analyses. We are trying to incorporate and integrate additional scRNA-seq data from studies with larger sample numbers and cell counts. We would like to clarify the detailed regulatory mechanisms of FLI1 in the tumor immune field.

## Conclusions

Taken together, our SMR results suggested that a higher DNA methylation level in the UTR region of FLI1 upregulated the expression of FLI1 and subsequently decreased BRCA risk. FLI1 displayed lower expression in BRCA tissues than in normal tissues, which likely resulted from the decrease of endothelial cells in tumor tissues compared with normal tissues. FLI1 was downregulated and might represent a potential biomarker contributing to the favorable prognosis of patients with BRCA. Thus, we concluded that FLI1 functions primarily as a protective factor against BRCA onset and development. FLI1 plays its fundamental role in the microenvironment of BRCA by targeting immune cells via regulating transcription activities of ligand and receptor genes in a cell-type-specific manner, which further exerts an effect on intercellular communication. We also first proposed a potential upstream regulatory mechanism of FLI1 in BRCA, namely CKMT2-AS1/PSMA3-AS1/DIO3OS-hsa-miR-324-5p-FLI1 axis. Nevertheless, we conclude these depending on the bioinformatic analysis, and it is better to validate these conclusions using biological experiments and large clinical trials in the future.

### Supplementary Information

**Additional file 1.**

**Additional file 2: Supplementary Figure 1**. Three-step SMR analysis prioritized FLI1 and mechanisms in BRCA. (A) Locus zoom plots show the genetic effects from BRCA GWAS and cis-eQTLs near FLI1. (B) The plot shows chromatin state annotations from REMC for different primary cells and tissue types. REMC, Roadmap Epigenomics Mapping Consortium.

**Additional file 3: Supplementary Figure 2.** Single-cell transcription analysis and regulatory network of FLI1. (A) UMAP projection of 99,532 cells, which were clustered into 21 clusters. (B) The dot plot shows FLI1 expression in different cell types. (C) Violin plots and dot plots of FLI1 expression in each cell type split by sample group. (D) The heatmap shows the activities of regulons of FLI1 and other cofactors in each cell type and is clustered according to the regulon activity. Colors from blue to red indicate low to high regulon activity. (E) Ridgeline plots and violin plots show the AUC values of the FLI1 regulatory network in each cell type integrating normal and tumor samples. N, normal. T, tumor. L, lymph node.

**Additional file 4: Supplementary Figure 3.** Crosstalk between all cells in BRCA. (A-B) Heatmaps showing summarizing the incoming (target) (A) and outgoing (secreting) (B) signal pathways of each cell group among all samples. (C) The stacked bar chart exhibiting the conserved and tumor or normal group-specific signaling pathway in cell communication. (D) The singling changes of T cells (left) or myeloid cells (right) in the normal group compared with the tumor group.

**Additional file 5: Supplementary Figure 4.** Crosstalk among all cells in BRCA. (A-B) Circle plots show the up or downregulated pathways of myeloid cells (A) or T cells (B) as target cells in communication with other cells in the tumor group compared with those in the normal group. (C) Bubble plots display the main signaling pathways mediating cellular interactions from endothelial cells (left), epithelial cells (middle), and B cells (right) to other cells. (D-E) Violin plots show the detailed expression of the genes related to the CXCL pathway (D) and the MHC I pathway (E) in each cell type in the different groups. (F) Bubble plots displaying the GO analysis results of the intersection genes of FLI1 target genes and ligand-receptor genes.

**Additional file 6: Supplementary Figure 5.** Expression and prognostic value of the ceRNA network of FLI1 in BRCA. (A) Negative expression correlations between predicted miRNAs and FLI1. (B-D) The expression of candidate miRNAs (B) and candidate LncRNAs (D) in BRCA and control normal samples from TCGA and the prognostic value of candidate miRNAs in BRCA (C).

## Data Availability

The datasets used and analyzed during the current study are available from the TCGA (https://portal.gdc.cancer.gov), GEPIA (http://gepia.cancer-pku.cn/), GEO (https://www.ncbi.nlm.nih.gov/geo/),TIMER (https://cistrome.shinyapps.io/timer) and CIBERSORT (https://cibersort.stanford.edu/).

## Abbreviations

BRCA: Breast cancer
ceRNA: Competing endogenous RNA
FDR: False discovery rate
GEO: Gene Expression Omnibus
GEPIA: Gene Expression Profiling Integrative Analysis
GO: Gene ontology
GSEA: Gene set enrichment analysis
GTEx: Genotype-Tissue Expression
ncRNA: Noncoding RNA
LncRNA: Long noncoding RNA
mRNA: Message RNA
miRNA: MicroRNA
KEGG: Kyoto Encyclopedia of Genes and Genomes
OS: Overall survival
TCGA: The Cancer Genome Atlas
TILs: Tumor-infiltrating lymphocytes
