# Supplementary figures and images for: Integrated analysis reveals FLI1 regulates the tumor immune microenvironment via its cell-type-specific expression and transcriptional regulation of distinct target genes of immune cells in breast cancer
Source: BMC Genomics. 2024 Mar 6;25:250. doi: 10.1186/s12864-024-10174-9 (PMC10916124; doi:10.1186/s12864-024-10174-9)

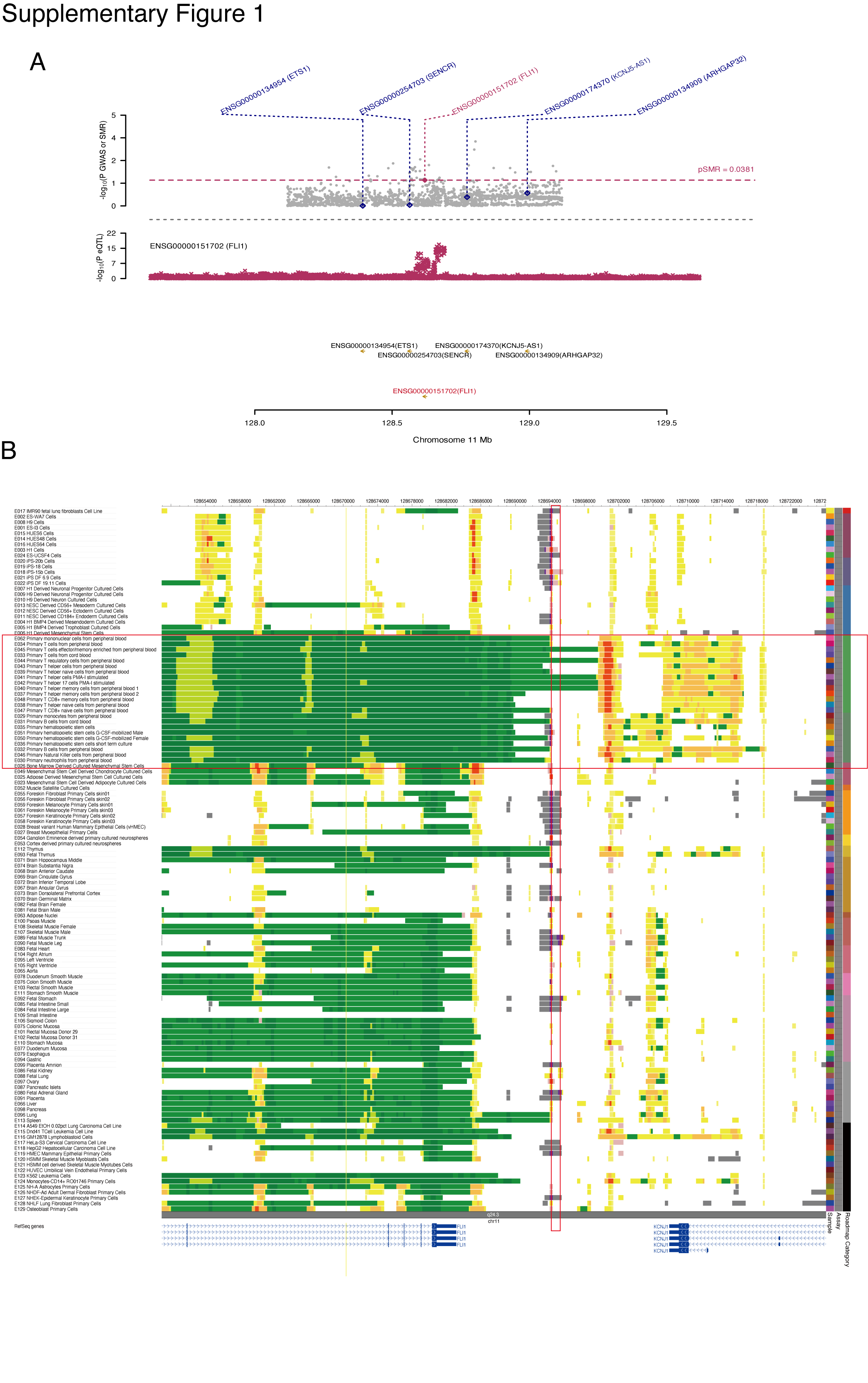

Supplement: Supplementary file 2 — Additional file 2: Supplementary Figure 1. Three-step SMR analysis prioritized FLI1 and mechanisms in BRCA. (A) Locus zoom plots show the genetic effects from BRCA GWAS and cis-eQTLs near FLI1. (B) The plot shows chromatin state annotations from REMC for different primary cells and tissue types. REMC, Roadmap Epigenomics Mapping Consortium. [file 12864_2024_10174_MOESM2_ESM.tif]

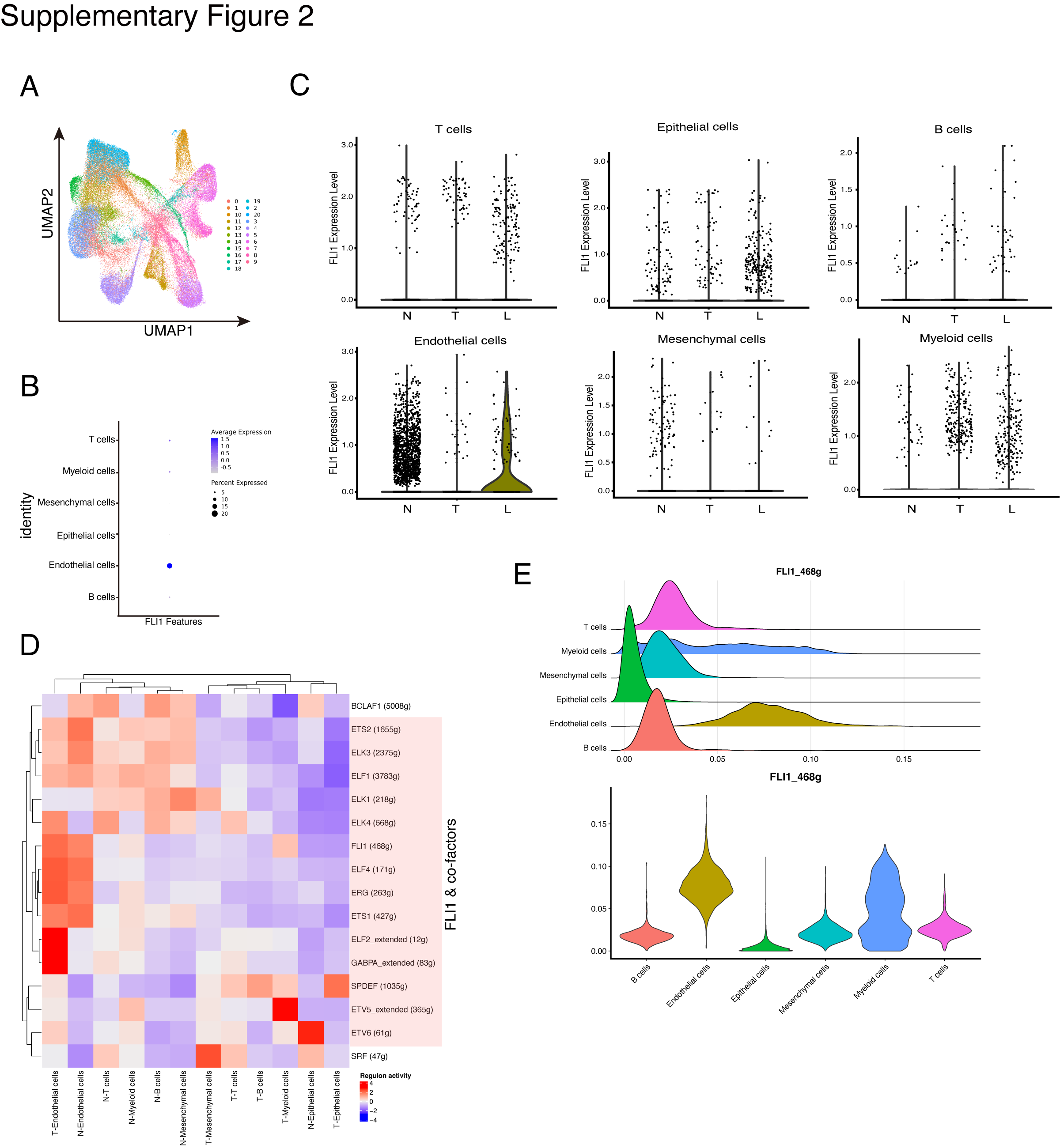

Supplement: Supplementary file 3 — Additional file 3: Supplementary Figure 2. Single-cell transcription analysis and regulatory network of FLI1. (A) UMAP projection of 99,532 cells, which were clustered into 21 clusters. (B) The dot plot shows FLI1 expression in different cell types. (C) Violin plots and dot plots of FLI1 expression in each cell type split by sample group. (D) The heatmap shows the activities of regulons of FLI1 and other cofactors in each cell type and is clustered according to the regulon activity. Colors from blue to red indicate low to high regulon activity. (E) Ridgeline plots and violin plots show the AUC values of the FLI1 regulatory network in each cell type integrating normal and tumor samples. N, normal. T, tumor. L, lymph node. [file 12864_2024_10174_MOESM3_ESM.tif]

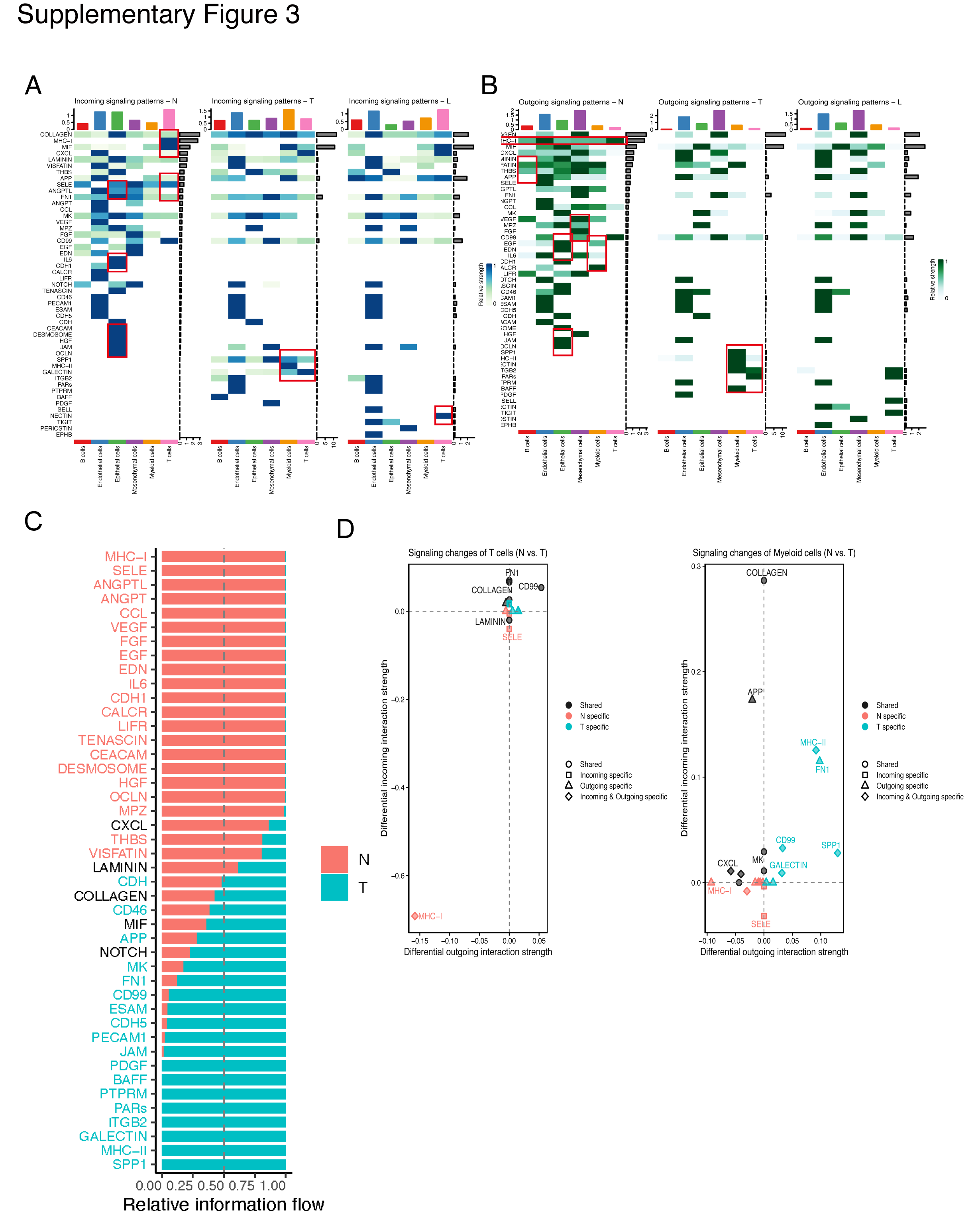

Supplement: Supplementary file 4 — Additional file 4: Supplementary Figure 3. Crosstalk between all cells in BRCA. (A-B) Heatmaps showing summarizing the incoming (target) (A) and outgoing (secreting) (B) signal pathways of each cell group among all samples. (C) The stacked bar chart exhibiting the conserved and tumor or normal group-specific signaling pathway in cell communication. (D) The singling changes of T cells (left) or myeloid cells (right) in the normal group compared with the tumor group. [file 12864_2024_10174_MOESM4_ESM.tif]

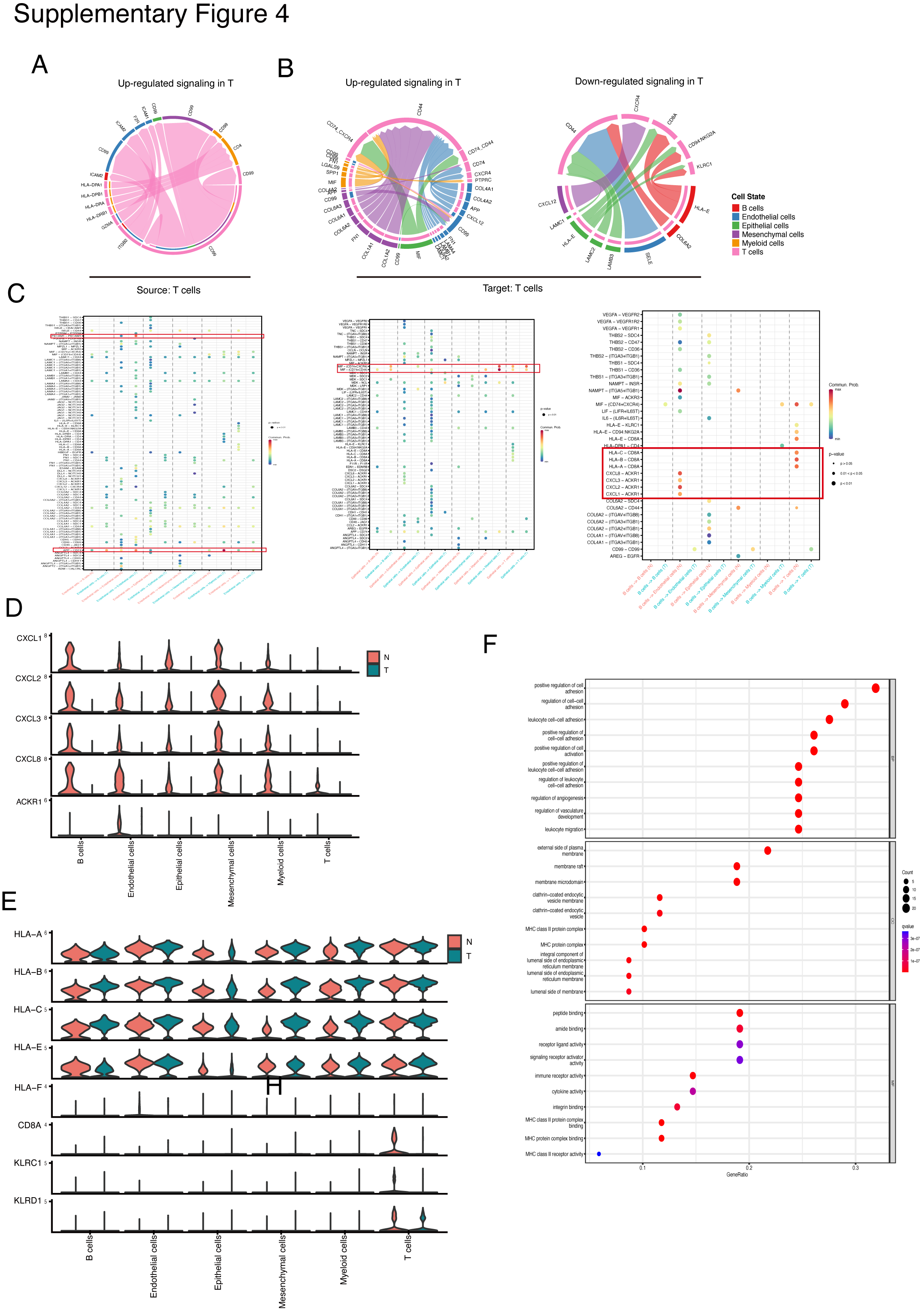

Supplement: Supplementary file 5 — Additional file 5: Supplementary Figure 4. Crosstalk among all cells in BRCA. (A-B) Circle plots show the up or downregulated pathways of myeloid cells (A) or T cells (B) as target cells in communication with other cells in the tumor group compared with those in the normal group. (C) Bubble plots display the main signaling pathways mediating cellular interactions from endothelial cells (left), epithelial cells (middle), and B cells (right) to other cells. (D-E) Violin plots show the detailed expression of the genes related to the CXCL pathway (D) and the MHC I pathway (E) in each cell type in the different groups. (F) Bubble plots displaying the GO analysis results of the intersection genes of FLI1 target genes and ligand-receptor genes. [file 12864_2024_10174_MOESM5_ESM.tif]

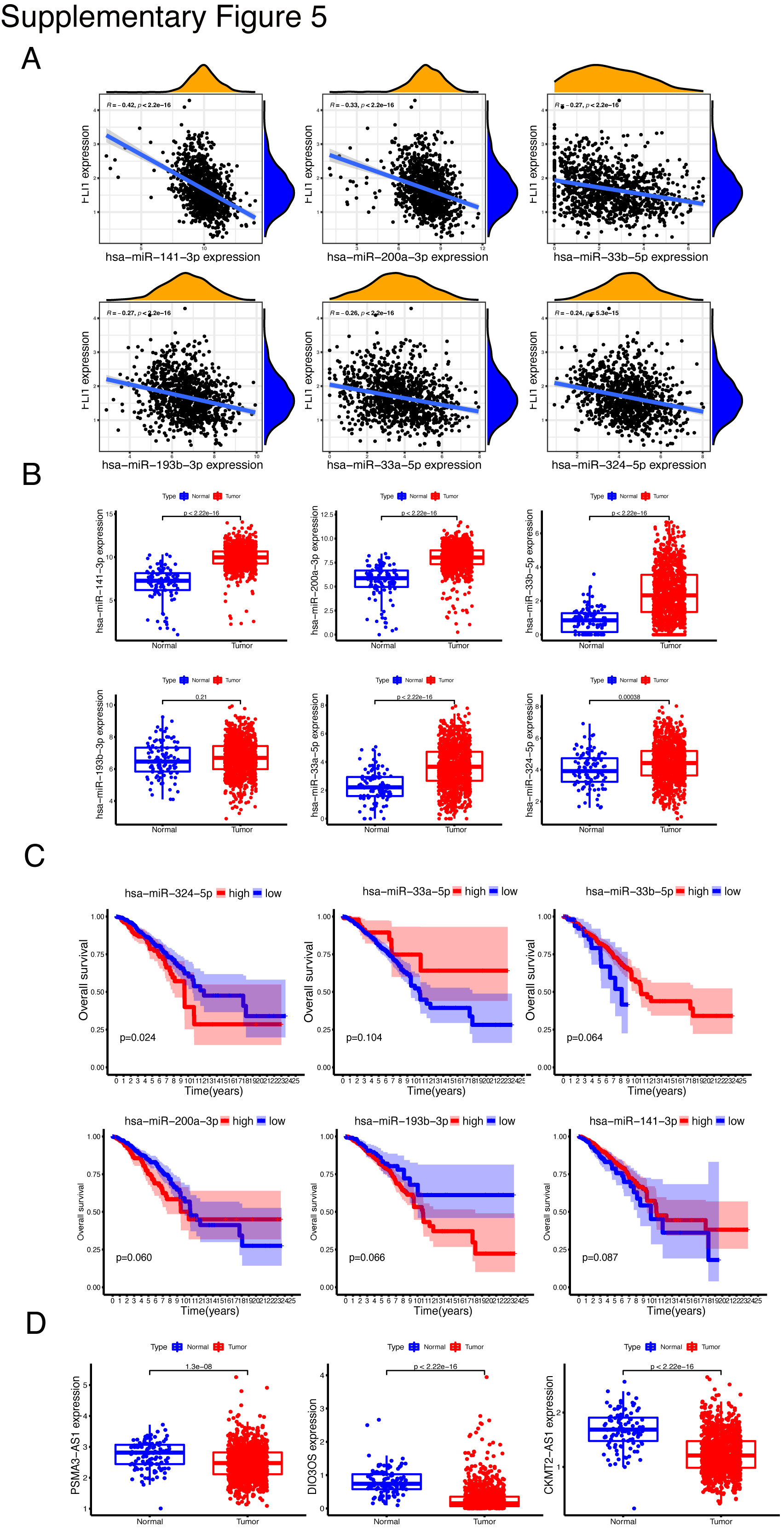

Supplement: Supplementary file 6 — Additional file 6: Supplementary Figure 5. Expression and prognostic value of the ceRNA network of FLI1 in BRCA. (A) Negative expression correlations between predicted miRNAs and FLI1. (B-D) The expression of candidate miRNAs (B) and candidate LncRNAs (D) in BRCA and control normal samples from TCGA and the prognostic value of candidate miRNAs in BRCA (C). [file 12864_2024_10174_MOESM6_ESM.tif]
